# Circulating tumor cells in breast cancer: A tool whose time has come of age

**DOI:** 10.1186/1741-7015-9-43

**Published:** 2011-04-21

**Authors:** Ramona F Swaby, Massimo Cristofanilli

**Affiliations:** 1 Department of Medical Oncology, Fox Chase Cancer Center, Philadelphia, PA, 19111, USA

## Abstract

Circulating tumor cells (CTCs) are isolated tumor cells disseminated from the site of disease in metastatic and/or primary cancers, including breast cancer, that can be identified and measured in the peripheral blood of patients. As recent technical advances have rendered it easier to reproducibly and repeatedly sample this population of cells with a high degree of accuracy, these cells represent an attractive surrogate marker of the site of disease.

Currently, CTCs are being integrated into clinical trial design as a surrogate for phenotypic and genotypic markers in correlation with development of molecularly targeted therapies. As CTCs play a crucial role in tumor dissemination, translational research is implicating CTCs in several biological processes, including epithelial to mesenchymal transition. In this mini-review, we review CTCs in metastatic breast cancer, and discuss their clinical utility for assessing prognosis and monitoring response to therapy. We will also introduce their utility in pharmacodynamic monitoring for rational selection of molecularly targeted therapies and briefly address how they can help elucidate the biology of cancer metastasis.

## Background

Affecting approximately 200,000 women in the United States alone, breast cancer is now recognized to be a heterogeneous disease comprised of several common different phenotypes [1]. Due to increased screening, awareness and consequent early detection, only approximately 5% of all breast cancer patients are initially diagnosed with incurable disease [2]. However, despite optimal local and systemic adjuvant treatment, 30-40% of patients diagnosed with curable breast cancer eventually die of recurrent disease [3,4]. Therefore, improved techniques to both detect and treat metastatic breast cancer are needed.

As early as the 1800's when Recaimer first coined the term "metastasis", circulating tumor cells (CTCs) have been postulated to be critical to the process [5,6]. The development of disseminated disease has been traditionally viewed as a sequential rather than concurrent process, i.e.- the disease initially occurs at the primary site, followed by local growth with eventual dissemination to distant sites. However, emerging data is challenging this theory [7,8]. In fact, the initiation of metastasis may be a relatively early event in tumor biology, underscoring the need to understand the significance of CTCs.

**Figure 1:**
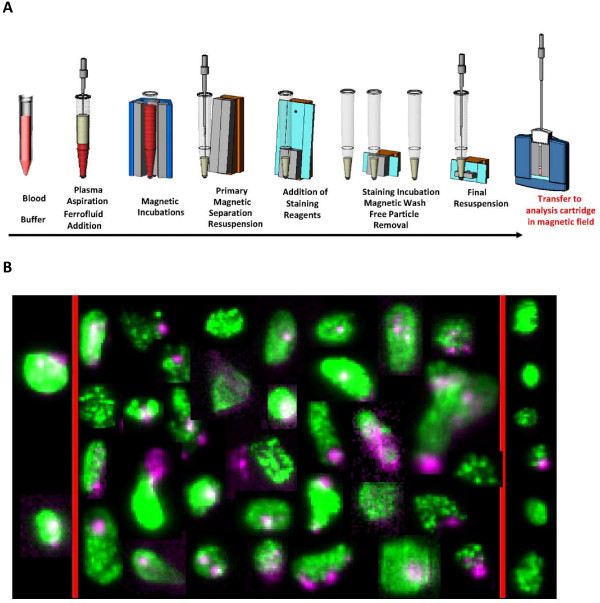
**(a) Schematic of Cell Tracks CTC separation system and (b) Detection of CTC**. a. Epithelial cells are isolated from peripheral blood using antibodies to Ep-CAM conjugated to magnetic particles. Cells are then analyzed to determine the number of CTC. b. Detection of CTC. Two intact CTC are shown in the left panel, 33 damaged CTC in the central panel, and 6 cellular fragments in the right panel.

CTCs are now generally defined as nucleated cells lacking CD45 and expressing cytokeratin [9,10]. Specifically, an antibody to the surface epithelial cell adhesion molecule (epCAM) identifies cells of epithelial origin circulating within the blood. Additionally, cytokeratin antibodies further distinguish CTCs as those that are not white blood cells (i.e. - CD45 negative) and select for carcinomas (i.e. - anti-CK8, anti-CK18, anti-CK19). Although multiple commercially available methods for isolating CTCs exist [11,12], the CellSearch™ system (Veridex Corporation, Warren, NJ) is the only FDA approved system for clinical use with reproducible results across many different laboratories. The CellSearch™ system has been fully described elsewhere [13]; but in summary, the system uses serum enriched for nucleated cells expressing epithelial-cell adhesion molecules, and fluorescently labels them for eventual detection by semi-automated fluorescence-based microscopy. (Figure 1) In this minireview the clinical trials reviewed all used the CellSearch™ system for CTCs isolation and reporting. This minireview will focus exclusively on the clinical utility of CTCs as it relates to patient care. We will first review the clinical trial data that validated CTCs ability to predict disease free and overall survival.

Additionally, more recent data comparing CTCs to both standard imaging for metastatic breast cancer and the newer [18F]fluorodeoxyglucose (FDG) positron emission tomography (PET)/computed tomography (CT) will be reviewed as well as new data correlating CTC burden as a surrogate for disease burden with the risk of developing thromboembolic disease.

### Clinical Utility

#### Predictive and Prognostic Capability

The first large, multi-institution, double-blind, prospective clinical trial evaluated the prognostic capability of CTCs in patients with metastatic breast cancer (MBC)[14]. One hundred and seventy-seven patients with measurable disease had CTCs tested prior to beginning a new palliative treatment regimen for progressive disease, followed by repeat assessment at first follow-up visit approximately 4 weeks later. This landmark trial prospectively identified a CTC cut-off level of ≥5 cells per 7.5 ml of blood to be a reliable identifier of patients at higher risk for disease progression and decreased survival from metastatic breast cancer. Regardless of histology, hormone receptor and HER2/neu status, or whether the patient had recurrent or de novo metastatic disease, those with <5 CTCs at baseline, and more importantly, at first follow-up after beginning a new treatment regimen, had superior progression free (PFS) and overall survival (OS) (7 vs 2.1 months [p < 0.001] and 10.1 vs >18 months, [p < 0.001], respectively). Additional CTCs assessment in this same patient cohort at essentially monthly intervals following first follow-up also confirmed improved PFS and OS for patients with <5 CTCs at any subsequent time point. PFS and OS for patients with <5 CTCs ranged from 5.6 to 7 months and 18.6 to >25.0 months, respectively, compared to those with ≥5 CTCs of 1.3 to 3.6 months PFS and 6.3 to 10.9 months OS (p = 0.001) [15].

Subsequent analysis of the eighty-three newly diagnosed patients undergoing first-line treatment for MBC in the above study confirmed the ability of CTCs to predict response to therapy and global outcome. At first follow-up assessment, median PFS for patients with <5 CTCs was 9.5 months, vs 2.1 months for those with ≥5 CTCs (p = 0.0057). Similarly, those with <5 CTCs had a median OS of 18 months, compared to just 11 months for those with ≥5 CTCs [16].

A similar analysis of the prognostic value of CTCs among newly diagnosed MBC patients prior to beginning first-line salvage therapy was performed in a large, retrospective single-institution study [17]. This study analyzed CTCs of 185 newly diagnosed MBC patients diagnosed between 2001 and 2007. As previously seen, patients with ≥5 CTCs at baseline had a greater than three-and-a-half fold increased risk of death, (HR = 3.64 [95% CI, 2.11-6.30, p < 0.0001]) compared to those patients with < 5 CTCs. The prognostic significance of CTCs was independent of choice of therapy (i.e. - chemotherapy with anthracyclines, taxanes, or both anthracyclines/taxanes, hormone therapy), and was also independent of hormone receptor status and HER-2/neu status. Interestingly, in this cohort, although the patient demographics were representative of the phenotypic characteristics of MBC patients in general, i.e. - approximately two-thirds of patients hormone receptor positive and approximately 20% HER-2/neu positive, greater than half of the patients had bone as their first site of metastatic disease. Upon multivariate analysis, patients with bone metastasis, compared to other sites of disease with ≥5 CTCs had an additional risk of death. (HR = 1.61; 95% CI 0.52-5.04 [p = 0.410]).

Additional analysis of CTCs and histologic breast cancer classification and phenotypes in a cohort of 517 MBC patients with either measurable or evaluable disease prior to commencement of new palliative treatment regimens has yielded interesting observations and hypothesis-generating information. Lobular histology and bony (but not visceral) disease burden were associated with higher numbers of CTCs [18]. In this study, a closer evaluation of the chemo-naïve HER-2/neu MBC patients treated with targeted HER-2/neu therapies, showed that almost all (13 of 14) demonstrated a decline in CTCs to <5, including patients with documented clinical and radiologic disease progression. At time of the report, the median OS in these 13 patients with CTCs decline had still not been reached, indicating that CTCs of less than five, as previously shown, correlated closely with superior prognosis, despite interval, episodic progression.

A summary of clinical trials reviewed that served to validate the predictive and prognostic capability of CTCs can be seen in Table 1.

**Table 1:** Summary of clinical trials reviewed that served to validate the predictive and prognostic capability of CTCs

Study Design	Primary Objective	**Prospective orRetrospective**	N	Line of therapy	Median PFS (months)	Median OS (months)
**1**. Multi-institution, double-blind, clinical trial of patients with MBC with measurable disease beginning a new therapy. CTCs assessed at baseline and at 1^st ^follow-up clinical visit, approximately 3-4 weeks after starting therapy.^14^	PFS, OS	Prospective	177	Any	7.0 vs 2.7; p < 0.001 baseline assessment 7.0 vs 2.1; p < 0.001 1^st ^follow-up	>18 vs 10.1; p < 0.001 baseline assessment >18 vs 8.2; p < 0.001 1^st ^follow-up

**2**. Multi-institution, double-blind, clinical trial of patients with MBC with measurable disease on treatment. CTCs assessed at 2^nd ^follow-up and subsequent follow-up clinical visits up to 9 months of follow-up. Sub-study of #1 above.^15^	PFS, OS	Prospective	177	Any	5.6 - 7 vs 1.3 - 6; p = n/a	18.6- >25 vs 6.3 - 10.9; p = 0.001

**3**. Multi-institution, double-blind, clinical trial of patients with MBC with measurable disease beginning a new therapy. CTCs assessed at baseline and 1^st ^follow-up clinical visit, approximately 3-4 weeks after starting therapy for the 47% of patients who were being treated with 1^st ^line palliative therapy. Sub-study of #1 above. ^16^	PFS, OS	Prospective	83	1^st ^line	9.5 vs 4.9, p = 0.0014 baseline assessment 9.5 vs 2.1, p = 0.0057 1^st ^follow-up	>18 vs 14.2, p = 0.0048 baseline assessment >18 vs 11.1, p = 0.0012 1^st ^follow-up

**4**. Single institution, retrospective analysis of a cohort of newly diagnosed MBC patients (either with *de novo *MBC or newly diagnosed recurrent MBC) who had CTCs assessed at the time of initial diagnosis of MBC between 2001 and 2007. CTCs assessed at baseline, prior to starting salvage therapy.^17^	OS	Retrospective	185	1^st ^line		28.3 vs 15, p < 0.001

**5**. Single institution assessment of a cohort of MBC patients prior to starting a new treatment regimen. CTCs assessment correlated with histology and phenotypic subtypes of breast cancer.^18^	Exploratory OS	Retrospective	517	Any		

CTC cut-off used in all of these clinical trials compared those patients with less than 5 CTCs (<5 CTCs), compared to those who had 5 or greater CTCs (≥5 CTCs) per 7.5 ml of whole blood.

#### CTCs and Imaging

In the prospective, longitudinal, multi-institutional trial described above, that demonstrated the ability of CTCs to predict PFS and OS, a nested retrospective study of 138 of the 177 enrolled patients was performed with the goal of comparing the predictive ability of CTCs assessment to standard imaging [19]. While there was no correlation between radiologic tumor burden and overall CTC levels, radiographic response was concordant with the established CTC cut-offs. Specifically, the almost two-thirds of patients who had evidence of radiographic response also had <5 CTCs at assessment, and an additional 16% had PD by both radiographic and CTCs (≥5) criteria. However, CTC responders, whether radiographic responders or non-responders, had similar significantly improved median OS. CTC non-responders, whether radiographic responders or not had worse outcome. Additionally, greater intrareader and interreader variability of interpretation of radiographic results compared to CTC enumeration was found (15.2% vs 0.7%). A separate retrospective study comparing the predictive capability of [18F]fluorodeoxyglucose (FDG) positron emission tomography (PET)/computed tomography (CT) imaging to CTCs enumeration in a cohort of 115 patients with MBC, again demonstrated CTCs superiority. As FDG-PET/CT is considered to be a promising new imaging modality in assessment of response for MBC patients [20-23], this radiographic assessment was performed at the same intervals as CTCs assessment, approximately 3 months after commencement of a new treatment regimen for progression of disease. In 102 evaluable patients, CTCs response correlated with FDG-PET/CT response 67% of the time, and in multivariate analysis was the most significant predictor of OS (p = 0.04)[24]. CTCs response, therefore, is most likely an accurate surrogate for radiographic response as well as those with stable disease and this is reflected in the superior clinical outcome associated with low number of CTCs. On the other hand, elevated CTCs, at anytime during the course of palliative treatment signals impending treatment resistance and progression of disease.

Of note, while CTC level does not correlate with radiographic measurable disease burden, CTC burden does correlate with extent of bony disease involvement. Relative to those without bone involvement, patients with multiple sites of disease including bony disease with ≥5 CTC had significantly worse outcome (p = 0.0008). This is in contrast to patients with minimal or no bone involvement, suggesting a potential biologic link between bone metastases and CTCs [25].

#### CTCs and Thromboembolic Disease

In a single institution, retrospective review, Mego and colleagues assessed CTCs level in 290 MBC patients prior to starting a new palliative treatment regimen. The presence of ≥1 CTCs, - the cut-off commonly employed in the adjuvant setting [14,26], was associated with a four-fold increase in thrombosis compared to patients who had no CTCs [27]. Although not statistically significant, patients with ≥5 CTCs were almost twice as likely to experience a thrombotic event (6.6 vs 11.6%, p = 0.076). When lines of therapy and extent of tumor burden were controlled for by multi-variate analysis, evidence of at least one CTC was still associated with greater than five-fold increase in the risk of thrombosis compared with those without any detectable CTC, thus confirming that CTCs, while not directly correlated with volume of disease burden, are a marker of increased morbidity that ultimately impacts mortality.

This observation indicates a potential role of clotting factors in the peripheral blood in some steps of the metastatic process. In fact, the environment in the bloodstream is highly unfavorable to tumor cells due to physical forces, presence of immune cells and death upon detachment (anoikis), which contributes to metastatic insufficiency [28]. Coagulation factors play an important role in metastasis and enhance breast cancer progression in animal models [29,30]. The binding of tumor cells to coagulation factors, including tissue factor, fibrinogen, fibrin and thrombin, creates an embolus and facilitates arrest in capillary beds followed by the establishment of metastasis [31]. This concept is further supported by a meta-analysis that showed anticoagulation therapy has beneficial effects on cancer patient survival [32]. However, the anti-metastatic effect of heparin is not a result of its anticoagulant activity but rather its ability to inhibit the interactions between some oligosaccharides present on tumor cells and P-selectin on platelets [33,34].

## Summary and Future Directions

Elevated CTCs, whether at baseline or at any time throughout the course of MBC are an ominous prognostic indicator. Whether by standard imaging studies and/or PET/CT imaging, elevated CTCs while on treatment, regardless of radiographic assessment, ultimately are predictive of an ineffective therapy. Similarly, low CTCs, especially while on treatment, predict for favorable survival as well as response to therapy. CTCs also reflect tumor biology as well as aspects of the host microenvironment as patients with elevated CTCs have an increased risk of developing thromboembolic disease. Taken as a whole, CTCs, while not directly correlated with volume of disease burden, are a marker of increased morbidity that ultimately impacts mortality.

Despite major advances in our understanding of cancer biology, we still lack detailed insight into the mechanisms of tumor establishment and dissemination. CTCs play a crucial role in tumor dissemination in relation to several biological processes, including epithelial to mesenchymal transition (EMT,) the process whereby epithelial cells lose cell-to-cell adhesion mediated by down-regulation of epithelial associated E-cadherin, and up-regulation of mesenchymal N-cadherin, allowing them to invade the extracellular matrix and migrate to a distant site [7]. The c-DNA of various genes such as Twist1, Akt2 and PI3Kα, associated with EMT and the stem cell marker, ALDH1, can be detected in CTCs. In patients with MBC not responding to the prescribed therapeutic regimen, the identification of EMT markers in the CTCs was considerably higher than in normal healthy individuals without a cancer diagnosis as well as patients with MBC who were responding to therapy [35].

**Figure 2:**
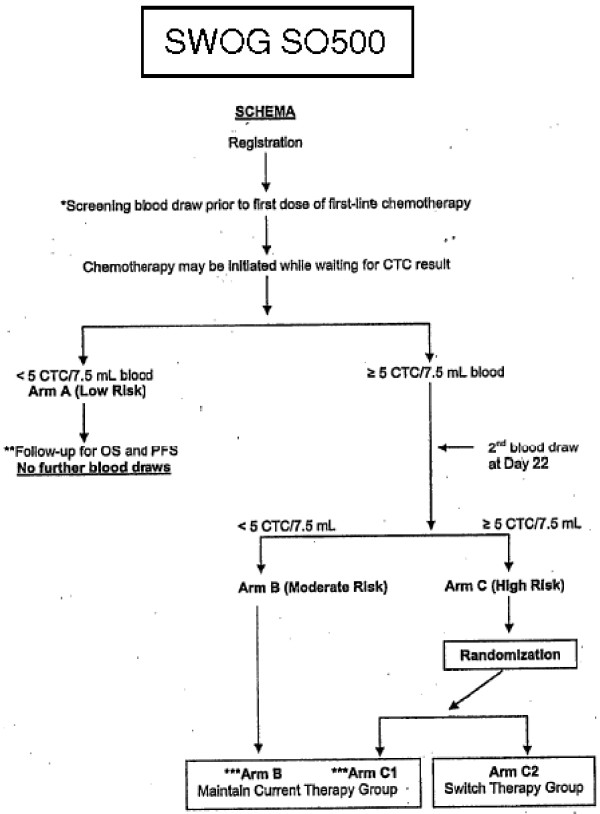
**Schematic of SWOG SO500**. A randomized phase III trial to test the strategy of changing therapy vs maintaining therapy for Metastatic Breast Cancer patients who have elevated circulating tumor cell levels at first follow-up assessment.

HER-2 amplification by fluorescent in situ hybridization (FISH) has been detected in CTCs, both concordant and discordant with the primary tumor phenotypic analysis. Interestingly, trastuzumab based therapy was administered to 4 of 9 patients with MBC in whom HER-2 positive CTC cells were detected, despite the primary site testing HER-2 negative by standard pathology techniques. Three of those four patients responded - one experiencing a complete response and two experiencing partial responses. This modest sample validates the hypothesis that the most relevant tumor cell population is the one that has proven ability to disseminate through the blood stream and may be the most representative population of metastatic disease [35]. Additionally, the possibility of collecting sequential blood samples for real-time monitoring of the efficacy of systemic therapies offers new possibilities to evaluate targeted therapies based on genomic profiling of CTCs and to improve the clinical management of patients with advanced disease [35-37]. This strategy is currently undergoing its first, large prospective, randomized validation study [38]. Patients who enroll on this study and have elevated CTCs will be randomized to either maintain therapy or switch treatments prior to standard re-staging by radiographic assessment (Figure 2).

## Conclusion

In conclusion, CTC assessment has been shown to be a repeatedly, strong and reliable predictor of outcome in metastatic breast cancer. It performs as reliably as imaging studies for assessment of response to therapy, and possibly more so. Although the mechanism is not fully elucidated, CTCs are a unique and heterogeneous cell population with established prognostic and predictive value in MBC particularly in defined subtypes of breast disease, and may related to bone biology in particular. In the era of biologically targeted therapies, molecular characterization of CTCs may hold the key to future pharmacodynamic assessment in drug development of MBC. Currently, most molecularly targeted biologic therapies are developed using the same toxicity assessment as traditional cytotoxic therapies - identifying the maximum tolerated dose. However, in the case of these newer molecularly targeted therapies, the maximum tolerated dose may not necessarily correlate with the most effective dose - i.e. receptor downregulation, protein phosphorylation, etc. The ability to repeatedly sample an accessible tumor population such as CTCs may allow selection of optimal therapies based on confirmed target delivery. Considerable effort is currently being directed at developing additional technologies to be able to reliably characterize the genomic and proteomic information contained within these cells. The full extent of CTCs utility has yet to be explored.

## Competing interests

The authors declare that they have no competing interests.

## Authors' contributions

RFS and MC were involved in drafting and editing the manuscript and MC has additionally given final approval for publishing. All authors read and approved the final manuscript.

## Pre-publication history

The pre-publication history for this paper can be accessed here:

http://www.biomedcentral.com/1741-7015/9/43/prepub
